# Large spatiotemporal variability in metabolic regimes for an urban stream draining four wastewater treatment plants with implications for dissolved oxygen monitoring

**DOI:** 10.1371/journal.pone.0256292

**Published:** 2021-08-24

**Authors:** Sarah H. Ledford, Jacob S. Diamond, Laura Toran

**Affiliations:** 1 Department of Geosciences, Georgia State University, Atlanta, Georgia, United States of America; 2 Environnement, Ville & Société (EVS UMR5600), Centre National de la Recherche Scientifique (CNRS), Lyon, France; 3 Department of Earth and Environmental Science, Temple University, Philadelphia, Pennsylvania, United States of America; Pacific Northwest National Laboratory, UNITED STATES

## Abstract

Urbanization and subsequent expansion of wastewater treatment plant (WWTP) capacity has the potential to alter stream metabolic regimes, but the magnitude of this change remains unknown. Indeed, our understanding of downstream WWTP effects on stream metabolism is spatially and temporally limited, and monitoring designs with upstream-downstream comparison sites are rare. Despite this, and despite observed spatiotemporal variability in stream metabolic regimes, regulators typically use snapshot monitoring to assess ecosystem function in receiving streams, potentially leading to biased conclusions about stream health. To address these important practical issues, we assessed the spatiotemporal variability in stream metabolism at nine sites upstream and downstream of four WWTPs in a suburban stream. We used one year (2017–2018) of high-frequency dissolved oxygen (DO) data to model daily gross primary productivity (GPP) and ecosystem respiration (ER). We found that GPP was 1.7–4.0 times higher and ER was 1.2–7.2 times higher downstream of the WWTPs, especially in spring when light was not limited by canopy shading. Critically, we observed that these effects were spatially limited to the kilometer or so just downstream of the plant. These effects were also temporally limited, and metabolic rates upstream of WWTPs were not different from sites downstream of the plant after leaf-out at some sites. Across sites, regardless of their relation to WWTPs, GPP was positively correlated with potential incident light suggesting that light is the dominant control on GPP in this system. Temporal windowing of DO to proposed regulatory monitoring lengths revealed that the violation frequency of water quality criteria depended on both the monitoring interval and start date. We conclude that spatiotemporal variability in metabolism and DO are crucial considerations when developing monitoring programs to assess ecosystem function, and that evidence of WWTP effects may only arise during high light conditions and at limited scales.

## Introduction

Stream metabolism is an integrative measure of riverine function that describes the capacity of streams and rivers to support ecosystem services like nutrient retention [1, 2] and carbon processing [3, 4]. This process-based measure is in contrast and complement to metrics of in-stream structure (e.g., biodiversity, [5]), and is increasingly viewed as a more direct appraisal of river management goals that often focus on river function [6–9]. Stream metabolism is routinely measured via the diel dissolved oxygen (DO) method [10], which allows simultaneous estimation of gross primary production (GPP), ecosystem respiration (ER), net ecosystem production (NEP = GPP + ER, where ER is negative by convention), and physical gas exchange (K). As in-situ DO sensors increase in reliability and decrease in cost [11–13], many state environmental agencies are considering DO as a proxy for ecosystem metabolism to assess and regulate stream health [14–16]. However, poorly constrained spatiotemporal heterogeneity in DO patterns limits the informed placement of regulatory measurements that intend to capture whole river system function [17, 18].

Multiple factors influence diel DO signals, from light to nutrient inputs, but their relative influence is poorly understood. High spatiotemporal variability in DO has been observed as a function of external drivers like light [19], frequency and timing of storms [20], and temperature [21, 22]. In contrast, the effect of nutrient (i.e., carbon (C), nitrogen (N), and phosphorus (P)) concentrations on DO (via changes to GPP and ER) is less clear [21, 23] and may only be evident when light and disturbance are not limiting to GPP [24]. Still, evidence suggests that nutrients associated with wastewater treatment plant (WWTP) effluent can influence DO concentrations primarily through increasing ER from added labile carbon [25–29]. In contrast, WWTP effects on GPP are equivocal [30–32], and light availability and hydrology appear to be the important drivers [33]. Still, these uncertainties surrounding WWTP effects to stream metabolism—particularly in how downstream effects to GPP and DO concentrations vary under contrasting environmental conditions—may challenge regulatory interpretation of DO signals as measures of ecosystem function.

While high temporal resolution DO monitoring has opened a new portal to understanding riverine function, it is clear that sampling frequency and window remain strong controls on subsequent inference [14, 34]. Many current regulatory protocols—typically created to address Total Maximum Daily Load (TMDL) requirements—do not require continuous monitoring, but may include short periods of diel data (days to weeks) or sampling at a consistent time of day [15]. Indeed, these TMDL-type measurements of water quality are likely more representative of ecosystem function, which can rapidly respond to regulatory changes, as opposed to ecosystem structure, which is driven by long-term trends [35]. This disconnect between sampling protocols and management goals may create tension and mistrust between lawmakers, regulatory agencies, and stakeholders [36, 37]. With predicted increases in stream temperatures [38], changing hydrologic regimes [39], and increased WWTP capacity [40] and their subsequent cumulative impacts on stream metabolism (e.g., [41, 42]), there is a clear need for adaptive management approach to DO sampling protocol to align with management goals [43].

Here, we focus on a suburban stream influenced by four WWTPs to address the growing need to understand connections between environmental drivers, disturbance, ecosystem function, and regulatory frameworks. Thus, the goals of this study were to (1) calculate the spatial and temporal variability of stream metabolism upstream and downstream of WWTP effluent inputs, and (2) evaluate the drivers of variation in stream metabolism response to WWTP inputs, and (3) examine the potential bias and mismatch between regulatory DO monitoring protocols and their desired outcomes. Spatial variability was assessed across ca. 1 km intervals upstream and downstream of the WWTPs, and temporal variability was assessed across seasons with periods of leaf-off, leaf-out, and warmer and cooler temperatures. We hypothesized that (1) WWTP discharge would amplify GPP and ER but that this effect would vary by season, that (2) WWTP effects on GPP would be modulated by reach-scale light-availability, with greater GPP effects under high-light conditions, and that (3) proposed environmental DO monitoring protocols such as 15-days or less time periods [44] would not capture important spatial and temporal heterogeneities in the system and therefore may bias interpretation of ecosystem function.

## Methods

### Site description

**Fig 1:**
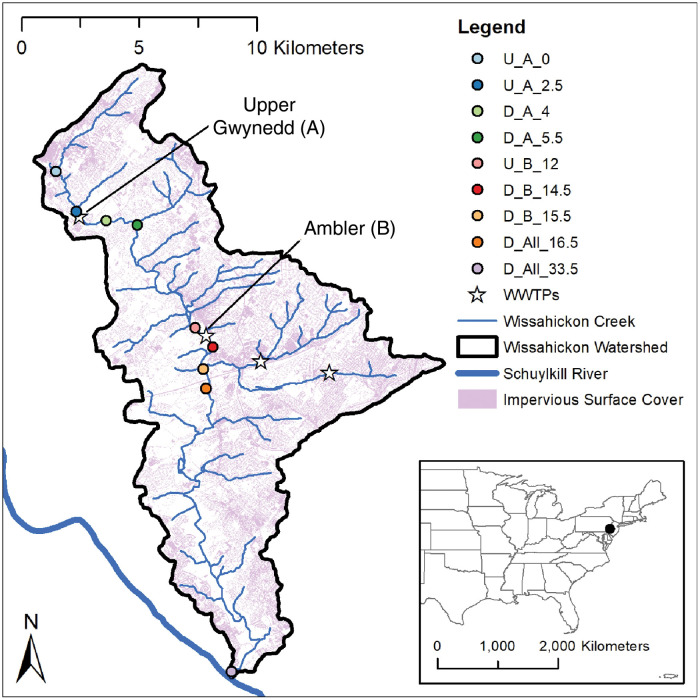
Site map. Wissahickon Creek watershed with locations of the nine monitoring sites and the four WWTPs. Each monitoring location site was equipped with a DO and depth logger. Site names refer to their location relative to a WWTP (U = upstream, D = downstream), the WWTP whose effect was analyzed (A = Upper Gwynedd, B = Ambler, or All = All WWTPs), and their distance downstream in km from the first site (0–33.5 km).

The Wissahickon Creek is an urban stream that runs from its source in the suburbs of Philadelphia, PA to its mouth at the Schuylkill River (Fig 1). The mainstem has a length of 43.5 km and watershed area of 165 km^2^ [45]. Approximately 50% of watershed land cover is impervious (Fig 1), leading to flashy discharge [45]. Despite urbanization, watershed preservation efforts have maintained a 15 m riparian buffer for 56% of the reaches on both sides and 25% of reaches on one side [46].

There are two WWTPs on the mainstem (Upper Gwynedd and Ambler) and two (Upper Dublin and Abington) on the largest tributary, Sandy Run, as the entire watershed is on sanitary sewers (Fig 1). In the headwaters, Upper Gwynedd WWTP effluent comprises 30–80% of approximately 0.1 m^3^ s^-1^ baseflow to the Wissahickon (Table 1). Downstream 10 km, Ambler WWTP contributes an additional 10–50% to the 0.3 m^3^ s^-1^ baseflow. The combined input from two WWTPs in Sandy Run is 40–50% of baseflow at the mouth of the tributary. Sandy Run contributes approximately 35% of the flow to the Wissahickon at the confluence. Therefore, depending on baseflow conditions, the four WWTPs comprise 25–90% of flow in the Wissahickon at the site just downstream of the Sandy Run confluence (site D-All-16.5; 0.7 m^3^ s^-1^ baseflow). Baseflow is approximately 1.5 m^3^ s^-1^ at the mouth (site D-All-33.5).

**Table 1:** Site information.

	Latitude	Longitude	Distance from nearest WWTP outfall or confluence (km)	Approx. baseflow discharge (m^3^ s^-1^)	WWTP contribution to baseflow^a^
U-A-0	40.20677	-75.29545	2.5 km US of A	0.002	—
U-A-2.5	40.19009	-75.28426	0.07 km US of A	0.09	—
D-A-4	40.18797	-75.27029	1.4 km DS of A	0.11	30–80%
D-A-5.5	40.18638	-75.25479	2.9 km DS of A	n/a	—
U-B-12	40.14707	-75.22565	0.6 km US of B	0.17	—
D-B-14.5	40.13957	-75.21677	0.7 km DS of B	0.32	10–50%
D-B-15.5	40.13123	-75.22167	1.8 km DS of B	0.32	—
D-All-16.5	40.12403	-75.21918	0.74 km DS of All	0.7	25–90%
D-All-33.5	40.01736	-75.20292	16.3 km DS of All	1.5	—

Locations of each monitoring site, including distance upstream (US) or downstream (DS) of the nearest WWTP (A = Upper Gwynedd, B = Ambler, and All = confluence with Sandy Run). Approximate WWTP contributions are included for sites immediately below a plant or confluence. Discharge measurements are from late summer.

^a^ Flow contribution varies based on time of year. Estimates range from high baseflows (low WWTP contribution) to low baseflows (high WWTP contributions).

WWTP effluent leads to increased nutrient concentrations downstream of each outfall, but concentrations diminish downstream (S1 Fig in S1 File). In the headwaters, nitrate (NO_3_–N) concentration increases by 10–20 mg L^-1^ downstream of the Upper Gwynedd WWTP, then returning to below 6 mg L^-1^. When the Ambler WWTP discharges, concentrations of NO_3_–N increase by 1–5 mg L^-1^. Along Sandy Run, NO_3_–N is attenuated over the 5.5 km downstream of the upper plant, with the lower plant adding minimal load to the stream [47]. At the confluence with Sandy Run, NO_3_–N is similar in both reaches so there is little or no change in concentration, but total dissolved phosphorous (TDP) increases by 0.1–0.2 mg L^-1^.

The entire mainstem of the Wissahickon and most of its tributaries are listed as impaired for aquatic life uses, attributed to high sediment and nutrient loads [48]. The EPA established TMDLs for NO_3_–N, ammonium, nitrite, phosphate, carbonaceous biochemical oxygen demand, and total suspended solids to address nutrients and siltation in the impaired reaches of the watershed. Given the assumed link between nutrients and DO, minimum DO standards set by the Pennsylvania Department of Environmental Protection (PADEP) were used as surrogate numeric criteria for the aquatic life use impairment caused by nutrients. PADEP has proposed continuous monitoring for periods of at least 15 days to identify diel swings that indicate excess productivity [44].

### Data collection

Data were collected year-round from April 2017 to May 2018 at nine sites on the Wissahickon Creek (Fig 1, Table 1). The sites are named for their location upstream (U) or downstream (D) of the uppermost WWTP (Upper Gwynedd = A), the second WWTP (Ambler = B), or all WWTPs (confluence with Sandy Run = All), and the distances to the nearest 0.5 km along the mainstem from the most upstream site (set at 0 km). The sites were accessed along a trail system open to the public and maintained by Wissahickon Trails, a non-profit land and water protection organization. Two of these sites were installed and maintained by the U.S. Geological Survey (USGS) in cooperation with the Philadelphia Water Department (PWD). The first site is Ft. Washington (USGS 01473900; D-All-16.5 in this paper), located in the center of the watershed, 16.5 km downstream of the upper-most monitoring site and 0.74 km downstream of the confluence with Sandy Run. The second site is Ridge Avenue (USGS 01474000; D-All-33.5 in this paper) at the mouth of the watershed. Both sites receive effluent from all four WWTPs and are used for regulatory monitoring. These two sites measure discharge at 15-minute intervals and DO at 30-minute intervals. The USGS/PWD does not collect DO data in the winter (early December–early March) at these sites, so we supplemented the data collection at these sites by installing a DO logger (PME MiniDOT, Vista, CA, USA) during this gap, overlapping for a week before and after the USGS removed their logger, measuring every 15 minutes.

The remaining seven measurement sites were strategically located to isolate effects of mainstem WWTPs and the Sandy Run tributary (Fig 1, Table 1). At WWTP A we monitored two upstream sites—at 2.5 km (U-A-0) and 0.1 km (U-A-2.5)—and two downstream sites—at 1.4 km (D-A-4) and 2.9 km (D-A-5.5). At WWTP B, we monitored one site 0.6 km upstream (U-B-12) and two downstream sites at 0.7 (D-B-14.5) and 1.8 km (D-B-15.5) downstream, both upstream of the confluence with Sandy Run. Differences in site spacing between the two WWTPs were made to avoid input of tributaries and to provide better access. The WWTP contribution to baseflow was estimated at sites immediately downstream of input where stage-discharge relationships were available. The contribution from WWTP is highest in late summer when baseflow is lowest.

At each site, we deployed optical DO sensors (Onset HOBO U26-001, Onset Computer Co., Bourne, Massachusetts, USA; YSI EXO2, Yellow Springs Instrument Co., Inc., USA; or PME MiniDOT). All loggers were calibrated before deployment in 100% saturated air and set to a 15-minute logging interval. The HOBO sensor has an accuracy of 0.2 mg L^-1^ below 8 mg L^-1^ of DO and 0.5 mg L^-1^ above 8 mg L^-1^ DO and a replaceable cap with a 6-month lifetime. The EXO2 sensor has an accuracy of 0.1 mg L^-1^ and included a wiper set to 1-hour intervals. The MiniDOT had an accuracy of 5% of the measurement (0.5 mg L^-1^ for 10 mg L^-1^ DO). Copper mesh with 0.15 mm opening was wrapped around the outside of the guard cage of each logger to minimize biofouling. No difference was observed between data from the various logger models. Loggers were housed in horizontal tile-drain tubing attached either to two pieces of rebar and elevated above the streambed, or to a cinderblock on the streambed. The seven DO loggers maintained by our group were calibrated before initial deployment and were removed and lab calibrated in the middle of deployment (October 31–November 2, 2017). Sensors were maintained and cleaned during monthly site visits.

At each site, we also obtained water level data, a requirement for areal-based metabolism estimates [49]. A HOBO Onset U13 water level logger collected water level data every 15 minutes at each site, along with a logger for barometric correction at the center of the watershed. USGS/PWD data were used for water level at those two sites.

Baseflow discharge data were collected at each of our seven sites using a handheld Acoustic Doppler Velocimeter (SonTek FlowTracker2, SonTek/YSI, San Diego, California). Baseflow measurements allowed estimation of channel cross sections at the site, which we supplemented with cross-sectional measurements 10 and 20 m upstream of the site. These three cross-sections were used to calculate average depth for the site. We linearly interpolated between sites to calculate reach-average depth. Baseflow velocity was calculated for each site using the discharge and cross-sectional channel area. Discharge and cross-sectional area data for the two USGS/PWD gauges were downloaded from the USGS National Water Information System database (waterdata.usgs.gov) and were used to calculate velocity.

### Sensor data processing

All data went through a manual quality control process, where we removed suspect or bad data from further analysis (see S2 Fig in S1 File for examples of data QAQC). We first removed DO data during storm periods when DO would drop to 0 mg L^-1^ due to sensor clogging (S2d Fig in S1 File). In addition, diel DO patterns were often interrupted during storms, resulting in days when metabolism could not be modeled (S2e Fig in S1 File). Hence, we removed data from metabolism calculations on days when there was both an abrupt change to the diel signal and a storm. We further smoothed single point anomalous DO noise with the average of the measurements before and after. For days where there was a clear diel signal, but noise between each individual measurement, 3-point smoothing was used (S2f Fig in S1 File). This smoothing was only needed at D-B-14.5 and was typically only needed during night measurements. This smoothing resulted in 0.35±0.01 mg/L (mean±se) of change from the original measurement across all smoothed data. If measurements were noisy with no underlying diel signal, data were removed (S2g Fig in S1 File). Data were also removed when site visits indicated drift (S2b Fig in S1 File), potential biofouling (S2h Fig in S1 File), or if the data were missing a diel signal (S2i Fig in S1 File). Full time-series of processed data shows the extent of the data sets at each site (S3 Fig in S1 File).

### Metabolism modeling

Stream metabolism was modeled using the one-station method [10] with the R package *StreamMetabolizer* [49]. Light data were modeled using the calc_light function and the latitude and longitude of each logger. The model was run with the Markov chain Monte Carlo state-space Bayesian framework accounting for both process- and observation-error with 1000 burn-in steps and 2000 saved steps. We constrained gas exchange (K600) estimates with tight priors, corresponding to the Bayesian *b_np_oipi_tr_plrckm*.*stan* model. We set the hyperprior (i.e., the prior distribution of the mean of K600 for all days) for each site as a lognormal distribution with mean and variance based on results from an initial run of the model using default values. We excluded model results where daily GPP was negative, where ER was positive, or where either of their 95% credible intervals contained 0 [25, 26]. We further ensured that all modeled metabolism days had an Gelman-Rubin statistic for both GPP and ER less than 1.05, indicating model convergence. We also conducted a visual check between modeled and measured DO and removed results where their correspondence was clearly poor (S4 Fig in S1 File). Across stations, we removed 19±5% (mean±standard error) of daily metabolism estimates between both methods, yielding 89 to 307 days of usable estimates for the monitoring period. On average, half of the estimates were removed for failing quantitative QAQC thresholds and half were removed by visual inspection. We visualized metabolism at each site using “metabolic fingerprints” [18], which are bivariate kernel density plots of ER vs. GPP that allow rapid assessment of their distributions.

### Potential incident light and seasonality

To estimate how light availability varied across our measurement sites and therefore how light may modulate WWTP effects, we calculated a potential incident light (PIL) metric at each site. PIL was estimated by comparing leaf-out and leaf-off Google Earth aerial imagery for a 300 m channel reach upstream of each DO logger. To simplify calculations and acknowledge within-reach variability, we separated each reach into 100-m longitudinal sections. For each 100 m section, we measured the visible channel area using the polygon tool for both leaf-off and leaf-out imagery. We then defined the PIL as the ratio of visible channel areas from leaf-out to leaf-off, where a site’s PIL was the mean of its PILs from each 100 m section within its 300 m upstream reach. We further calculated average stream width as the leaf-off channel area divided by the reach length.

Seasons were defined based on a combination of changes in PIL due to leaf cover, changes in stream temperature, and changes in DO. The end of spring 2017 (April 20, 2017) was defined by average leaf-out in the Philadelphia area during this year, reported by the National Phenology Network [50]. There was no observable change to DO concentrations from leaf-off at any sites, so summer and fall were lumped into a single season. We defined the end of fall as the time when average weekly temperatures upstream of WWTP influence (at U-A-2.5) remained below 4°C—December 10, 2017. Likewise, the end of winter was defined as the time when average weekly temperatures upstream of WWTP influence remained above 4°C—February 10, 2018. The remainder of the time series (through May 7, 2018) was defined as spring as there was no temporal change in DO concentrations during leaf-out in 2018 as there was by the end of April the previous year.

### WWTP effects on metabolism

To test our first hypothesis that WWTP discharge would stimulate GPP and ER, we compared their rates upstream and downstream of WWTPs using the non-parametric Kruskal-Wallis rank sum test using kruskal.test in R version 4.0 [51]. This tests the null hypothesis that median metabolic rates upstream and downstream of WWTPs differ, given that they have similar distributions. For all statistical analyses done in this paper, p < 0.001 is defined as highly significant, 0.01 > p > 0.001 is defined as moderately significant, and 0.05 > p > 0.01 is defined as weakly significant.

Because we were also interested in seasonal differences in metabolism, we conducted these upstream-downstream Kruskal-Wallis tests for each season, as described above. For the Kruskal-Wallis tests of upstream-downstream differences in metabolism, we constrained our sample set to days when both upstream and downstream sites had data. We further calculated an amplification metric (GPP_amp_ or ER_amp_), which is the ratio of median metabolic rate downstream to upstream. As for the Kruskal-Wallis test, these amplification metrics were calculated for each day when both upstream and downstream sites had data, and were summarized across each upstream-downstream pair and season.

We evaluated our second hypothesis that WWTP effects on GPP would be modulated by reach-scale light-availability using linear regression. We regressed annual and seasonal mean daily GPP and annual and seasonal mean DO amplitude (a proxy for GPP, [52]) by site and season against multiple metrics representative of WWTP influence, including PIL, width, depth, baseflow velocity, and average nutrient concentrations. Significance of linear models was calculated with an F-test on the regression model using the same p-value thresholds described above using the fitlm function in Matlab.

### Spatiotemporal variability in metabolism and DO

We evaluated our hypothesis that proposed sampling routines would not capture important spatiotemporal variability along the network in several ways. We first assessed within-site temporal variability in metabolic estimates by comparing seasonal differences with Kruskal-Wallis rank sum test using the kruskalwallis() function in Matlab and the same statistical thresholds described above. If metabolism varied across seasons, we interpreted this as evidence of temporal variability. We then evaluated spatial variability by comparing seasonal and annual metabolic rates across all possible site pairs using Kruskal-Wallis rank sum tests within each season using the same methodology.

Finally, to more directly evaluate spatiotemporal influences on DO-based monitoring protocol, we sliced our DO time series into windows of consecutive 3, 7, 15, and 30- day periods and calculated their daily DO minima and mean, metrics relevant to management (e.g., [15, 44]). We chose these sampling windows to capture the most likely sampling schemes of managers and because they were representative of possible sampling windows (S1 Table in S1 File). At each site, we considered every possible windowed period in this analysis: for example, for a 15-day window, we would evaluate DO metrics from the 15 days from April 12–26, April 13–27, and so on. We allowed data gaps of up to 3 days in our definition of “consecutive”. For example, we would consider DO measurements on March 13, 15, 18, 19, and 22 to be consecutive, and would calculate three 3-day windows starting on March 13, 15, and 18, respectively. We then assessed the intra- and inter-site variability in these metrics depending on time of year and window length by counting the number of times a particular sampling period would have resulted in a violation of DO water quality criteria from PADEP (mean < 6 mg L^-1^) and the EPA (mean minimum < 5 mg L^-1^; [53]). While both of these criteria focus on a 7-day averaging period, we averaged across the entire window length (i.e., 3, 7, 15, or 30 days) to assess how averaging length changes the violation rate. Moreover, because we consider every possible windowed period, there would be no difference among violation counts across varying window lengths greater than 7 days if we used the 7-day average method.

## Results

The results are reported in three sections, one discussing spatial trends, one discussing likely spatial drivers, and one discussing temporal trends, including sampling intervals. However, spatial and temporal trends are intertwined and the some of the same figures are referenced in each section.

### Spatial variability in physical characteristics and metabolism

Average depth along the Wissahickon increased downstream, with a maximum baseflow depth of 0.48 m at D-All-16.5 (Table 2). Similarly, stream width increased downstream, reaching a maximum of 35 m near the mouth of the stream. Stream velocity was more variable, showing the heterogeneity of stream morphology and subsequent impact on flow. U-A-0 and D-B-14.5 were both downstream of reaches with pools and had very low velocities while D-A-4, D-All-16.5 and D-All-33.5 were all in runs with higher velocities.

**Table 2:** Physical site characteristics.

Site	Average PIL (% ± se)	Average width (m ± se)	Average depth (m)	Baseflow velocity (m/s)
U-A-0	57.4 ± 15.6	3.6 ± 0.7	0.26	0.008
U-A-2.5	29.8 ± 4.9	7.9 ± 1.3	0.24	0.044
D-A-4	42.2 ± 6.2	9.8 ± 0.6	0.35	0.103
D-A-5.5	24.6 ± 7.5	13.9 ± 1.2	0.34	0.014
U-B-12	30.3 ± 3.3	14.5 ± 1.4	0.23	0.076
D-B-14.5	41.0 ± 6.7	16.3 ± 1.6	0.45	0.009
D-B-15.5	40.3 ± 1.9	18.9 ± 1.2	0.40	0.025
D-All-16.5	46.9 ± 2.2	17.7 ± 1.9	0.48	0.101
D-All-33.5	n/a	34.8 ± 0.9	0.42	0.123

Potential incident light (PIL) and reach physical characteristics at each site.

Potential incident light (PIL) was highly variable, driven by variations in the riparian canopy cover that extends over the stream (Table 2). U-A-0 is the only reach without mature riparian vegetation, with 88% sun over the 100 m just upstream of the logger. Downstream reaches (D-B-15.5 and D-All-16.5) have higher PIL as the stream widens. There is a shift in bedrock geology downstream of D-All-16.5 where the stream enters a deep bedrock valley with banks incised up to 37 m and bank slopes around 45°. For this reason, we excluded D-All-33.5 site from subsequent light-based analyses as our PIL method is unable to account for these shading effects.

**Fig 2:**
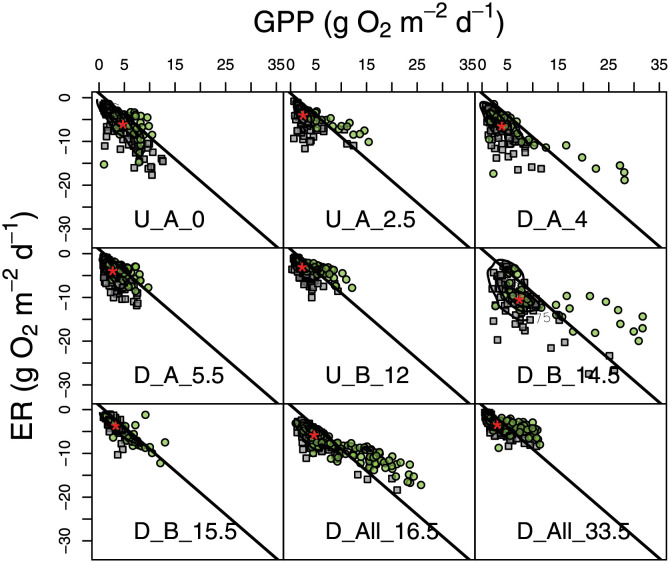
GPP vs. ER. Metabolic fingerprints at each site. Spring samples are in green circles and samples from the other seasons are in grey squares, red stars indicate the median values for the entire period, and lines indicate 1:1 relationship between GPP and ER where points above this line indicate net autotrophy and points below indicate net heterotrophy.

Stream metabolism and physical gas exchange varied along the Wissahickon at the annual scale (Fig 2, Table 3), with no clear longitudinal trend (Pearson correlations: r_dist,GPP_ = 0.016, r_dist,ER_ = 0.156). Indeed, the entire observed range of GPP and ER was found between sites only 2.5 km apart: median annual GPP and ER reached their network minima of 2.3 and -3.1 g O_2_ m^-2^ d^-1^ at U-B-12 and reached their maxima of 7.4 and -10.5 g O_2_ m^-2^ d^-1^ at the next site downstream, D-B-14.5 (Table 3). K600 ranged from a median of 4.8 d^-1^ at D-B-15.5 to 11.0 d^-1^ just upstream at D-B-14.5 (Table 3). ER and K600 at each site were poorly correlated (R^2^ = 0.00–0.28; S5 Fig in S1 File), suggesting that model results were not subject to strong equifinality [49]. Moreover, metabolism estimates span nearly the full range of depths for each site, although U-A-0 and D-B-15.5 were somewhat under-sampled at low flows (S6 Fig in S1 File). Median annual GPP and ER were highly correlated across sites (R^2^ = 0.92), but ER was consistently higher than GPP leading to negative NEP (“net heterotrophy”) across sites (Table 3). NEP was the most negative at sites just downstream of WWTPs (D-A-4 and D-B-14.5), and the least negative at U-B-12 and D-B-15.5.

**Table 3:** Annual metabolism estimates.

Site	n (d)	K600 (d^-1^)	GPP (g O_2_ m^-2^ d^-1^)	ER (g O_2_ m^-2^ d^-1^)	NEP (g O_2_ m^-2^ d^-1^)
U-A-0	153	6.9 ± 0.3	4.7 ± 0.2	-6.1 ± 0.3	-1.4±0.2
U-A-2.5	178	5.9 ± 0.3	2.5 ± 0.2	-4.0 ± 0.2	-1.2±0.2
D-A-4	134	6.1 ± 0.4	3.9 ± 0.4	-6.5 ± 0.3	-2.0±0.3
D-A-5.5	253	7.1 ± 0.3	2.7 ± 0.1	-4.0 ± 0.1	-1.3±0.1
U-B-12	263	10.2 ± 0.3	2.3 ± 0.1	-3.1 ± 0.1	-0.5±0.1
D-B-14.5	119	11.0 ± 0.6	7.4 ± 0.6	-10.5 ± 0.4	-2.1±0.5
D-B-15.5	89	4.8 ± 0.3	3.3 ± 0.3	-3.7 ± 0.3	-0.1±0.2
D-All-16.5	307	4.8 ± 0.2	4.7 ± 0.3	-5.8 ± 0.2	-0.6±0.2
D-All-33.5	294	6.3 ± 0.2	3.0 ± 0.1	-3.4 ± 0.1	-0.2±0.1

Median annual stream metabolism across sites with standard errors.

**Fig 3:**
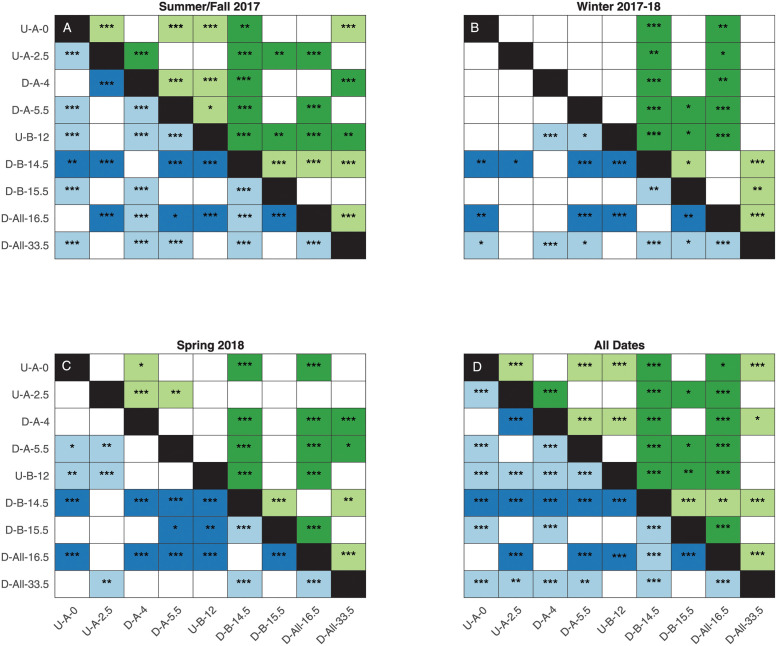
Site differences in GPP and ER. Kruskal-Wallis test results for GPP (top right of the black line, green) and ER (bottom left of the black line, blue) for (a) summer/fall 2017, (b) winter 2017–18, (c) spring 2018, and (d) all dates. The color of the cell indicates the direction of change in metabolism. Dark cells indicate downstream metabolism was higher than upstream for that pair (or upstream was lower than downstream), while light cells indicate downstream metabolism was lower than upstream for that pair (or upstream was higher than downstream). Asterisks indicate the p-value of the relationship, with p < 0.001 indicated with ***, 0.01 > p > 0.001 indicated with **, and 0.05 > p > 0.01 indicated with *. P-values are listed in S1-S4 Tables in S1 File.

We observed large spatial variability in stream metabolism across sites, as indicated by large heterogeneity in paired Kruskal-Wallis tests (Fig 3). There is spatial variability in GPP and ER even prior to significant WWTP influence: U-A-0 and U-A-2.5 differed on annual basis and in summer/fall. On an annual basis, sites were more likely to be different than similar (72% of site pairs were different for GPP and 75% for ER).

### Drivers of metabolism change

**Fig 4:**
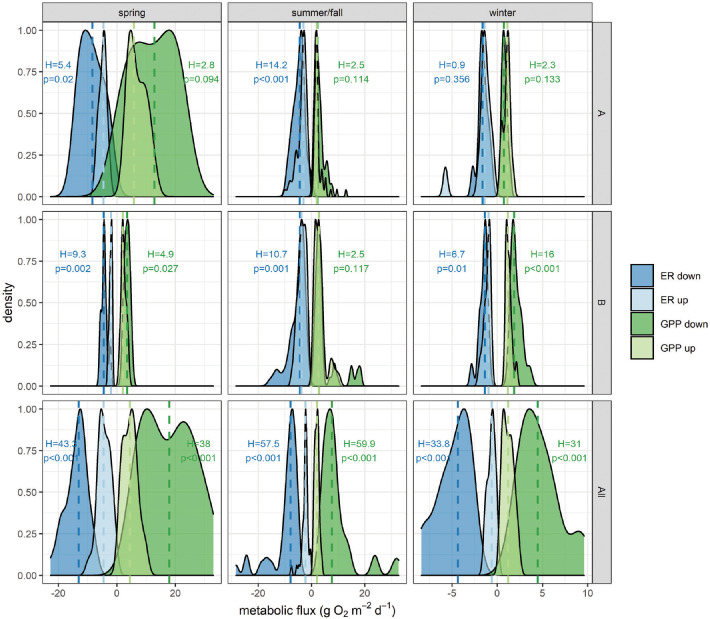
Upstream to downstream metabolism comparison. Kernel density plots of metabolic fluxes immediately upstream (light colors) and downstream (dark colors) of WWTPs (A, B, and All) across seasons (spring, summer/fall, and winter) for days when both upstream and downstream sites had measurements. D-B-15.5 is used as the upstream site for All. Medians of each distribution are shown in vertical dashed lines. Also shown are Kruskal-Wallis test results with the p-value, where p-values less than 0.05 indicate differences in median values. Note changing x-axis scales and that some extreme values were cutoff to improve visualization.

GPP and ER were amplified at sites immediately downstream of WWTPs and at site D-All-16.5, which is downstream of the Sandy Run confluence that transports effluent from two additional WWTPs (Fig 4). Across groups (i.e., WWTP A, B, and All), GPP amplification effects do not change across seasons (F_256_ = 2.45, p = 0.08), whereas ER amplification effects were strongly significant (F_256_ = 8.44, p < 0.001). Across groups, amplification effects were larger in spring (GPP_amp_ = 3.2±0.2, ER_amp_ = 3.8±0.6, mean±se) compared to summer/fall (GPP_amp_ = 2.4±0.2, ER_amp_ = 2.6±0.3). GPP_amp_ was intermediate in winter (GPP_amp_ = 2.7±0.3), but ER_amp_ was maximized, albeit with high variation (11.1±4.0). At WWTP A, GPP distributions were never different between upstream and downstream at p = 0.05, although ER distributions differed weakly in spring and strongly in summer/fall (Fig 4). All other upstream-downstream comparisons differed across sites and seasons except GPP for summer/fall at WWTP B. The differences were always strongly significant below the confluence with Sandy Run. Spring showed the greatest metabolic amplification across sites, which generally became weaker across seasons.

**Fig 5:**
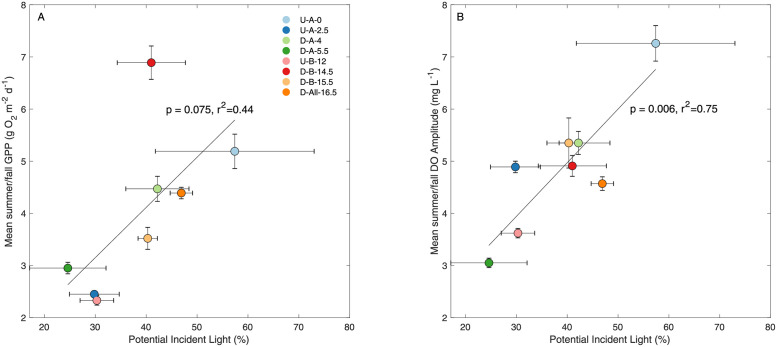
Linear regressions of PIL and metabolism. Linear regressions between PIL and (a) mean summer/fall GPP and (b) mean summer/fall DO amplitude. Error bars represent the standard error. Sample sizes to calculate mean values ranged from 31 to 196 estimates.

PIL explained 44% of the variance in mean summer/fall GPP, almost weakly statistically significant (p = 0.075), and 75% of the variance in mean summer/fall DO amplitudes at a moderately statistically significant level (p = 0.005; Fig 5). Metabolism rates were also compared to physical stream characteristics (Table 2) and average nutrient concentrations, but no statistically significant linear relationships were found.

### Temporal impacts: Seasonal variability and sampling window

**Fig 6:**
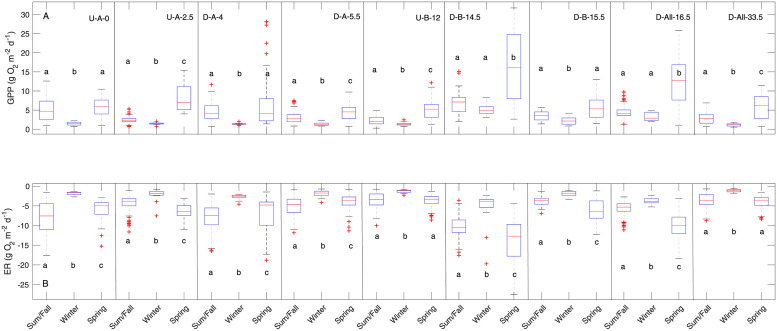
Boxplots of seasonal GPP and ER. Boxplots of (a) seasonal GPP and (b) seasonal ER. Site are organized from upstream (left) to downstream (right). All sites exhibited strong seasonal differences (p < 0.001) according to the Kruskal-Wallis rank sum test and results are shown with letters indicating differences among seasons. Full statistical results are in S5 Table in S1 File.

There was clear temporal variability in stream metabolism within sites reflected in seasonal differences, and this variability differed between GPP and ER (Fig 6a and 6b). GPP differed strongly among all seasons for four sites (U-A-2.5, D-A-5.5, U-B-12, and D-All-33.5) while the others had the same rates during some seasons- typically summer/fall and spring (at U-A-0, D-A-4, and D-B-15.5). ER rates varied more than GPP among seasons, with all sites except U-B-12 and D-All-33.5 having different rates each season (Fig 6b). For both GPP and ER, winter showed the least variability across sites, as over half of the sites had the same metabolism (56% of pairs were the same for GPP and 53% for ER, Fig 3) while spring was generally the most variable (Figs 2 and 3). Spring periods were often associated with positive NEP (“net autotrophy”; see points above the 1:1 line in Fig 2), although median spring NEP was the same as winter NEP at all sites except D-All-33.5 (S7 Fig in S1 File).

**Fig 7:**
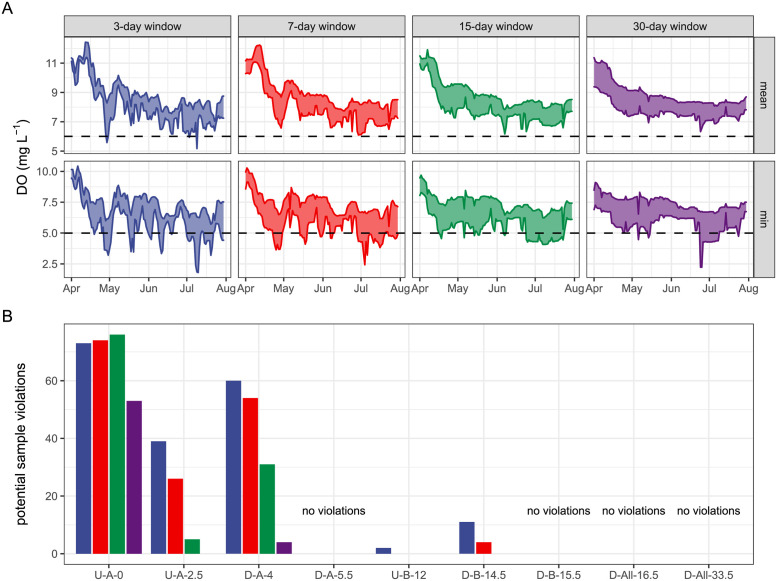
Windowing effects on DO violations. Effects of sampling window size and sampling start date on DO inference. (a) Pooled across-site variation (ribbons indicate interquartile range) in estimates of mean daily DO and mean daily DO minima (rows) as a function of moving window size (columns) for the critical period of April–August 2017. Dashed lines indicate chronic water quality standards for DO. (b) Effect of sample window size (colors match “A”) on the count of potential violations (both mean and minimum) of water quality standards by site.

We further found a clear effect of sampling window length and starting date on inferred DO means and mean minima (Fig 7). For example, there is an across-site difference of 2±1.5 mg L^-1^ (mean±se) in observed DO metrics if a 7-day sampling campaign began on May 5 versus May 15. Indeed, the campaign that started on May 15 would observe DO violations at three of the nine sites, but one that started on May 5 would observe none (compare daily minimum violations for the 7-day window in the month of May; Fig 7a). Moreover, there was large (~2 mg L^-1^) across site variability in DO metrics independent of sampling window or start date (ribbon widths in Fig 7a). This across site variability was greatest during the shift from leaf-off to leaf-out near the end of April and in mid-July.

Across sites, we observed that averaging over long windows generally reduced the likelihood of crossing chronic DO water quality standard thresholds (based on averages of mean and minima along the sampling window; Fig 7b), but these patterns were only evident for upstream sites. Downstream sites did not exceed DO water quality thresholds during our study period, further suggesting that site location and temporal window are strong controls on water quality assessment. Windows for the average minimum DO (Fig 7a) illustrate the importance of considering whether short term effects are significant. Longer windows show fewer violations of mean values indicating that DO effects are not sustained for long periods.

However, some regulations count a single day below 5 mg/L as a violation. At our sites, only three have more than 15 days of DO below 5 mg/L across the days where metabolism was modeled (S6 Table in S1 File). At D-All-33.5 despite a nearly full year record, there were no violations. Our long-term record shows how a shorter sampling interval can reduce single-day violations. For sites with fewer than 15 days below 5 mg/L, most short-term records will not capture any violations. For a site like U-A-0 with 59 days below 5 mg/L, a longer sampling interval will capture more violations. Monitoring for 30-days could result in capturing 8 to 14 days below 5 mg/L (S8 Fig in S1 File). That drops to around 4 to 8 days for a 15-day window and only 1 to 4 days with a 7-day window.

## Discussion

### WWTPs amplify metabolism locally, modulated by light

We observed clear support for our first hypothesis that stream metabolism is amplified downstream of WWTP effluent (Figs 3 and 4, Table 3). Critically, however, this amplification effect was local, both in space and time, driven by light availability, in line with our second hypothesis (Fig 5). Indeed, along the temporal dimension, WWTP effects on GPP and ER were greatest in spring, when light is abundant prior to leaf-out, with approximate doubling or quadrupling of rates. These effects tended to decrease throughout the year, more so for GPP than ER (Figs 2 and 3), suggesting that WWTPs may continuously increase respiration throughout the year, but only spur GPP during periods when light is not limiting. The largest amplification effects were observed downstream of WWTP B, supporting previous work in this system that also found higher DO-based ER downstream of WWTP B than WWTP A [33]. These seasonally-driven amplification effects are in direct alignment with analogous studies from Germany [41] and Spain [32], implying a common headwater stream ecosystem response to nutrient subsidies [54].

We found further support for our hypothesis that the differences in GPP downstream of WWTPs are spatially driven by light-availability. Perhaps unsurprisingly (cf. [21, 55, 56]), potential light availability was the best predictor of GPP across sites in the summer/fall, regardless of their relation to a WWTP (Fig 5). This relationship could further have been improved by accounting for variation in light attenuation with depth [19] as depths across sites can vary by a factor of two (Table 2). Likely because of this strong spatial control on light availability, we observed that amplification effects were longitudinally limited, with rates returning to pre-WWTP levels after a few km (Fig 3). This contrasts with previous work that showed increasing effects with downstream distance [32]. This difference may be explained by differences in light patterns in between the two systems: light availability the Wissahickon does not unambiguously increase in a downstream direction (Table 2) as opposed to the clear increase in [32] and as is conceptually expected in the River Continuum Concept [57]. This may be in part due to the small spatial extent of this study, but may be further due to a dominant urban land cover that induces geomorphological heterogeneities in local stream structure. These observations support contemporary revisions to eutrophication models of lotic waters, where nutrient additions alone are often insufficient to stimulate GPP without ample light availability [23, 58]. At locations where the largest portion of water is sourced from effluent (D-A-4 and D-A-5.5, Table 1), the metabolic rates are not the highest, even in summer when baseflow is lowest, showing that even in these highly impacted streams, understanding variations in light is of first-order importance.

The longitudinal limit to amplification effects implies that WWTP effects are not additive; to our knowledge no other study has examined an urban system with successive WWTPs like the Wissahickon. Indeed, spatial and temporal attenuation in WWTP amplification leads us to suggest that WWTPs act as discontinuities in stream metabolic networks, acting as control points whose effects become smoothed and integrated downstream [59]. Hence, WWTPs may create nodes of high metabolic variability in the network that turn on during critical biogeochemical windows of opportunity (*sensu* [54]; compare upstream-downstream variances in Fig 4). Sub-daily changes in effluent quantity and quality combined with hydrologic and biogeochemical diel cycles create highly complex systems that are difficult to piece apart [33, 47].

In addition to light availability, there are several plausible explanations for the large spatiotemporal diversity in metabolic regimes in this small stream network (Figs 3 and 6). Indeed, magnitudes of net heterotrophy vary considerably across WWTP-impacted systems [26, 30, 31], highlighting the importance of other drivers. Importantly, storms can reduce GPP [60] due to scouring of autotrophs from benthic surfaces [20, 61] and by reducing light availability in the water column from turbidity effects [62]. Metabolic recovery time following storm events varies [63, 64], depending on season (vis-à-vis light and temperature) and disturbance magnitude [22, 65], all of which may be modulated by local hydraulic and geomorphic conditions leading to divergent reach behavior and response to WWTPs. However, a qualitative evaluation of the largest storm during our monitoring period (S9 Fig in S1 File) shows equal and rapid recovery of DO signals along the network both upstream and downstream of WWTPs, indicating similar metabolic response and recovery to storm events across sites. Variable storm response therefore does not appear to be a major driver of differences in metabolic regime in the Wissahickon.

Another important source of variability in metabolism likely derives from geomorphic heterogeneity in this small stream network. For example, the first site downstream of all four WWTPs (D-All-16.5) sustained a relatively high summer GPP but was further than any other downstream site from an effluent source. Previous work suggests that the effluent signature arriving at this site from the WWTPs on Sandy Run has already been attenuated [47]. However, there is a distinct shift in bed material between Sandy Run (sand) and D-All-16.5 (gravel, cobbles, and boulders). That shift in substrate combined with a widening of the stream (Table 2) allowed for large, filamentous algae mats to grow in spring, with benthic chlorophyll *a* concentrations as high as 1400 mg m^-2^ at the center of the stream in April 2018 (S10 Fig in S1 File). Further, this increase in chlorophyll *a* was not observed in sites immediately downstream of WWTPs, which had spring mid-channel maximum concentrations of 330 to 460 mg m^-2^. These observations provide support for the notion that geomorphological factors, through their capacity to adjust fine sediment accumulation, control potential metabolic rates [66].

### Regulatory monitoring may bias metabolic and dissolved oxygen realities

High frequency data over long periods, like those presented here, can improve understanding of factors leading to low DO in streams. However, such data sets can be discouraged in the regulatory environment where a single violation or short-term average can lead to an impairment designation [36]. This is a particular problem when stream assessment is viewed as a binary descriptor of meeting or not meeting criteria [67]. We found a snapshot of minimum DO is not representative of the magnitude of stream processes that regulators want to infer, as our third hypothesis stated. This is best highlighted with the observation of the highest mean DO in spring (Fig 7) along with the highest GPP (Figs 2 and 6), which illustrates a clear mismatch between what the regulators indicate they want to control (eutrophication from high GPP) and their proposed mechanism for monitoring (mean DO). With this contrast between data and regulations, our results invite a reconsideration of the numeric stream criteria intended to regulate stream ecosystem processes. Given the rapidly growing and more spatiotemporally representative DO datasets [14], there is clearly an opportunity for adaptive management of numeric criterial to better understand when to monitor conditions that create hypoxia or algal blooms [20, 68] and how to use data to determine underlying stream processes [37, 43] and move beyond binary criteria.

Adapting regulations to the heterogeneity we observed means considering both temporal and spatial variations, moving away from a “one size fits all” approach. The first step of this approach is to have a clear goal for the monitoring program, then linking the monitoring program to that goal or adapting when a different goal is posed. If elimination of chronic hypoxic events (low DO) is the goal, then results presented here suggest that nighttime monitoring of DO during late summer (July-August) will provide the best indicator of status (Fig 7). But if reduction of GPP is the goal, then agencies should have multiple monitoring points downstream of potential hot spots in springtime. In general, the dynamic nature of DO and stream metabolism results suggests an adaptive management approach that can shift focus from instantaneous and binary designations of impaired or not impaired with respect to DO and instead use functional ecosystem management at seasonal and annual scales.

Our dataset provides some guidance for adapting temporal DO monitoring scales. For regulations that allow for temporal averaging, we suggest a monitoring periods of 30 days because inferences drawn from shorter periods are highly dependent on the window length (Fig 7). Indeed, windowing of the long-term data set presented here across different monitoring intervals showed significant variation in assessment of violations depending on both start date and length of the window (Fig 7 and S8 Fig in S1 File). Importantly, longer monitoring periods increase the chance of observing regulatory violations, further demonstrating the growing potential for mismatches between regulatory criteria and the ecosystem processes they are designed to protect. Overall, we observed that although long term monitoring periods identified more violations of binary criteria than short term data, the average behavior over longer intervals reduced violations. Hence, we caution averaging over long periods as it may obscure temporal deviations that can be used to understand causes of impairment.

Although monitoring across seasons is preferable for whole ecosystem understanding (Fig 6), shorter periods are more commonly used by regulators, so care should be taken to account for how the time of year of the monitoring start date affects expected results. For example, we saw large across-site variation about the mean in the spring before leaf-out (Fig 2), and leaf-out periods tended to be more representative of the annual average (Fig 3). So, depending on if average behavior, spatial variability, or maximum rates is the desired result, summertime or springtime data may be more informative. Still, we advocate for an adaptive management approach to data collection that accounts for how sampling can influence interpretation of ecosystem behavior.

Increased spatial characterization of stream DO will complement the growing temporal perspective and should inform a selection framework for regulatory monitoring locations. Currently, regulatory monitoring is conducted at two sites on the Wissahickon: D-All-16.5 and D-All-33.5. Our addition of seven more sites, in particular those sites upstream of the WWTPs, illustrated a large reach-scale heterogeneity in metabolism rates across this system. Higher GPP rates (Fig 2) and higher potential violations of threshold criteria (Fig 7 and S6 Table in S1 File) were observed upstream of the WWTPs and variations between GPP at downstream sites suggest that additional factors besides effluent need to be evaluated to assess stream metabolism. When comparing sites, it is important to evaluate potential incident light, water depth, and distance from nutrient sources and try to include variation in these factors in sampling design. Our study highlighted the importance of including the potential incident light for comparing monitoring sites. In addition, we recommend estimating GPP where possible, as DO amplitude did not always align with GPP (Fig 5). Thus, the heterogeneity of urban streams needs to be considered when making stream metabolism measurements and designing a monitoring program.

## Conclusions

We observed high spatial and temporal variability in urban stream metabolism predominately driven by WWTP input and seasonal and longitudinal variation in light availability. There is a persistent and clear increase in metabolism downstream of WWTPs, but this response is spatially limited, with rates returning to pre-WWTP within a few km. During leaf-out periods, however, 75% of the variability of mean diel DO amplitude can be explained by light, and there is a smaller response downstream of plants. This inter- and intra-site variability in DO and metabolism is not captured by typical regulatory monitoring programs, resulting in an incomplete picture of the ecosystem functioning of the stream. In other words, regulatory DO measurements fail to capture the ecosystem function that management aims to control. We therefore advocate for an adaptive approach to regulatory criteria for DO that can be matched to a specified management goal and updated in accordance with rapidly increasing ecosystem knowledge.

## Supporting information

S1 File (DOCX)
