# Supplementary material for: Large spatiotemporal variability in metabolic regimes for an urban stream draining four wastewater treatment plants with implications for dissolved oxygen monitoring
Source: PLoS One. 2021 Aug 24;16(8):e0256292. doi: 10.1371/journal.pone.0256292 (PMC8384190; doi:10.1371/journal.pone.0256292)
Supplement: S1 File — (DOCX) [file pone.0256292.s001.docx]

**S1 File**

**
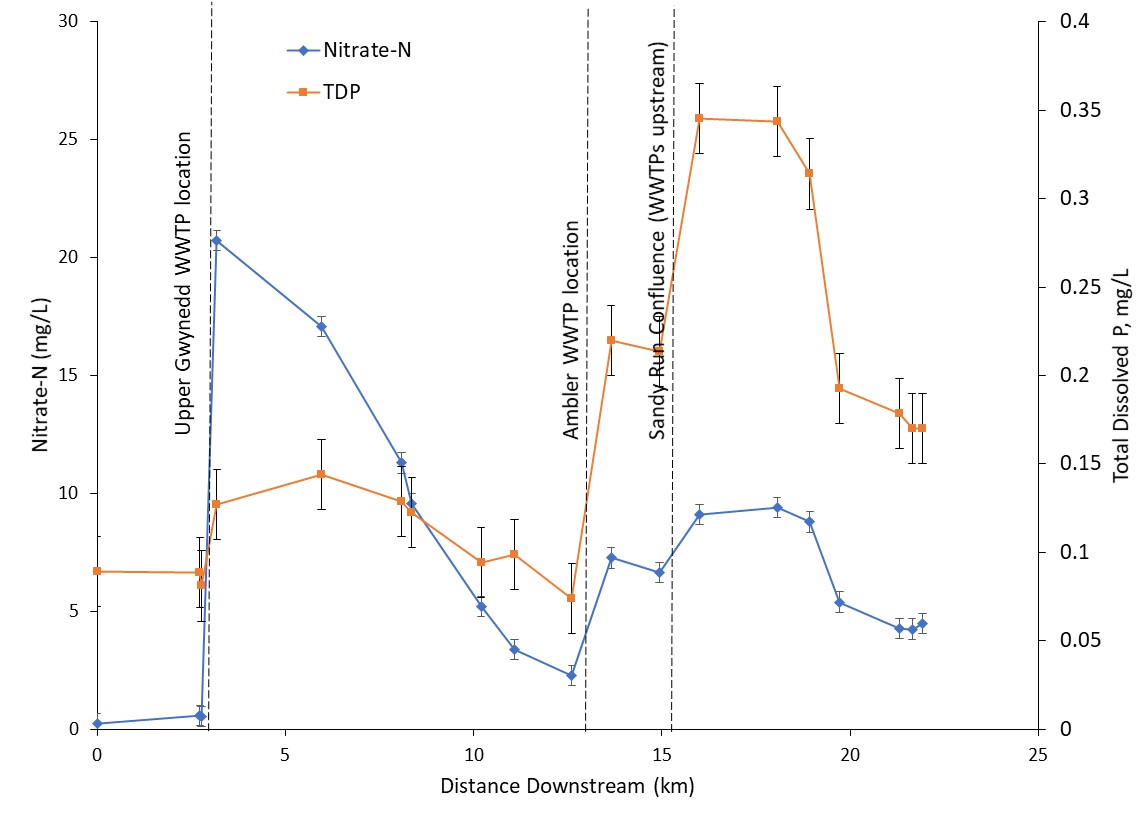
**

**S1 Fig. Longitudinal nutrient concentrations.** Nutrient concentrations from longitudinal sampling on 7/1/2016 shows increase in nitrate and total dissolved phosphorous (TDP) immediately downstream of each WWTP and Sandy Run confluence (with upstream WWTPs). Dilution downstream occurs then increases for each WWTP input. Nitrate analyzed by the authors using ion chromatography; TDP analyzed by the authors using ICP-OES.


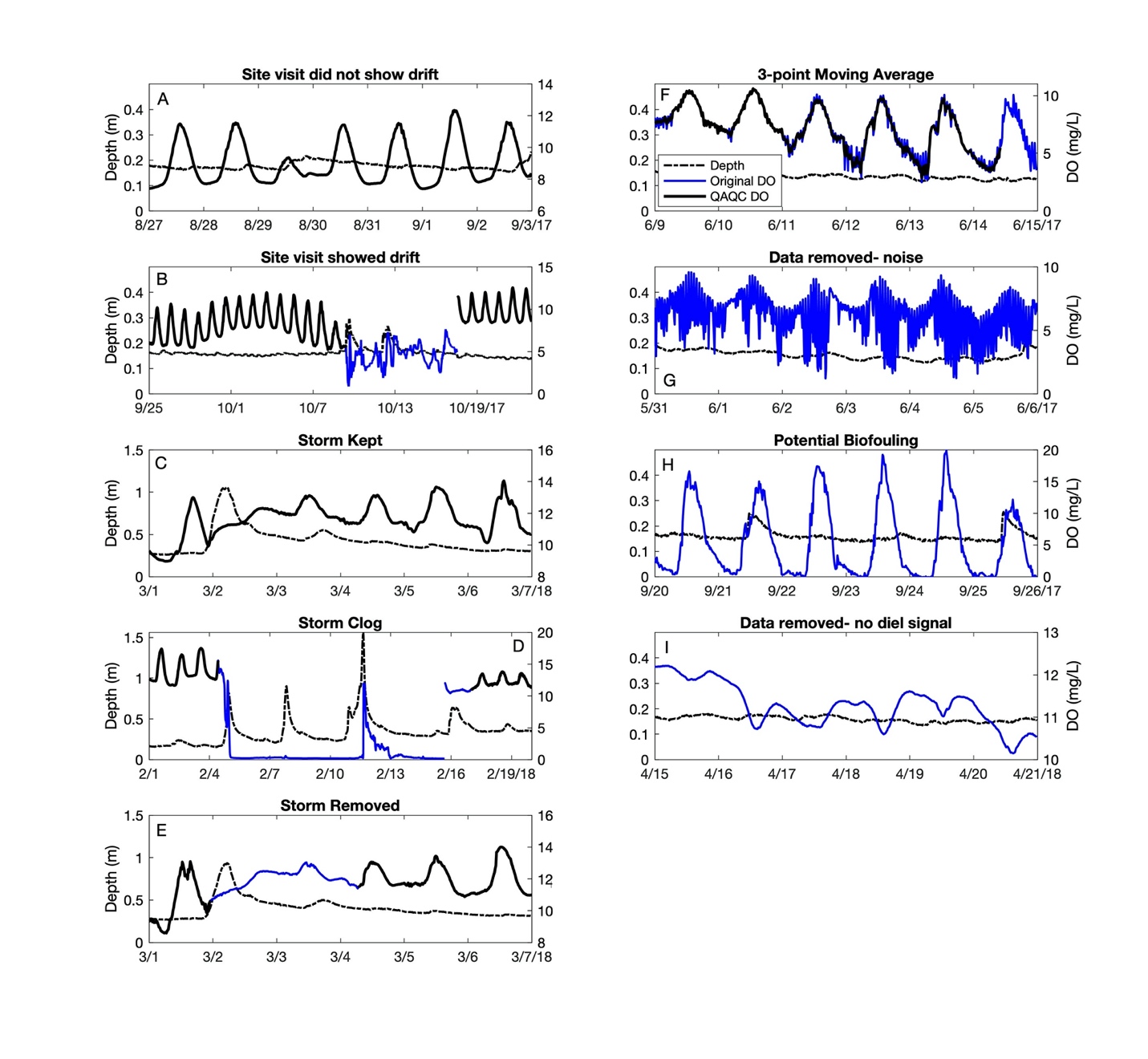


**S2 Fig. DO QAQC examples.** Examples of high-quality DO data (A and C) and poor-quality data (B and D-I) as determined by the QAQC process. In all figures, blue lines indicate measured DO that was removed from further analysis because of poor quality, while the solid black line indicates DO data that was retained for further analyses. The dashed line indicates depth. A and B are from U-B-12 and show the difference between a site visit where there was no change after cleaning and download, so no indication of drift or other issues (A, with the site visit on 8/30/17) vs. a site visit where there were quality control issues with data before the download, so data were retroactively removed until the point where there was a clear diel signal (B, site visit on 10/18/17). C-E show typical responses to storms. C (from site D-A-4) shows a storm where the diel signal was retained, so no data were removed; D (from site D-B-14.5) shows an instance where the logger was buried during a storm, which was cleared out during the next site visit on 2/15/18; and E (from site D-A-5.5) shows a period where the storm interrupted the diel signal, so that period of data was removed, until the diel signal returned. F and G are two periods of data from D-B-14.5, with F showing a period where a 3-point moving average was used to remove noise while retaining the underlying diel signal, while G shows a period where noise was too great to remove. H (from site U-A-0) shows a period of data that was removed because of concerns of biofouling, identified by the DO signal fluctuating between 0 mg/L at night and very high (up to 20 mg/L) DO during the day. I (from site D-B-15.5) shows a period that was removed because of the lack of diel DO signal. Note: the ranges of x and y-axes change between panels.


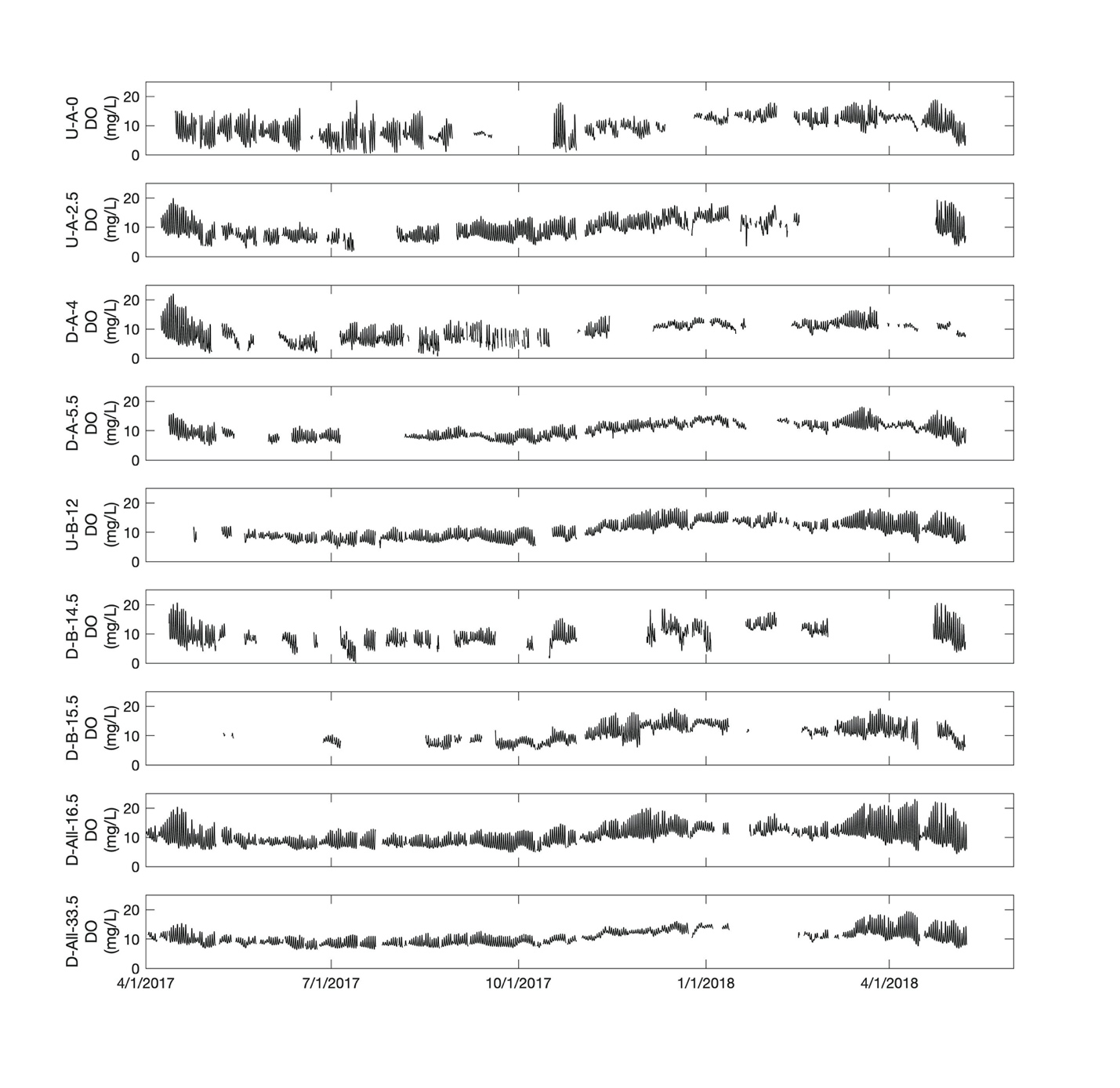


**S3 Fig. Complete DO time-series.** The complete QAQC-approved DO signal from each site after poor-quality data were removed. The y-axis scale is held constant across panels.


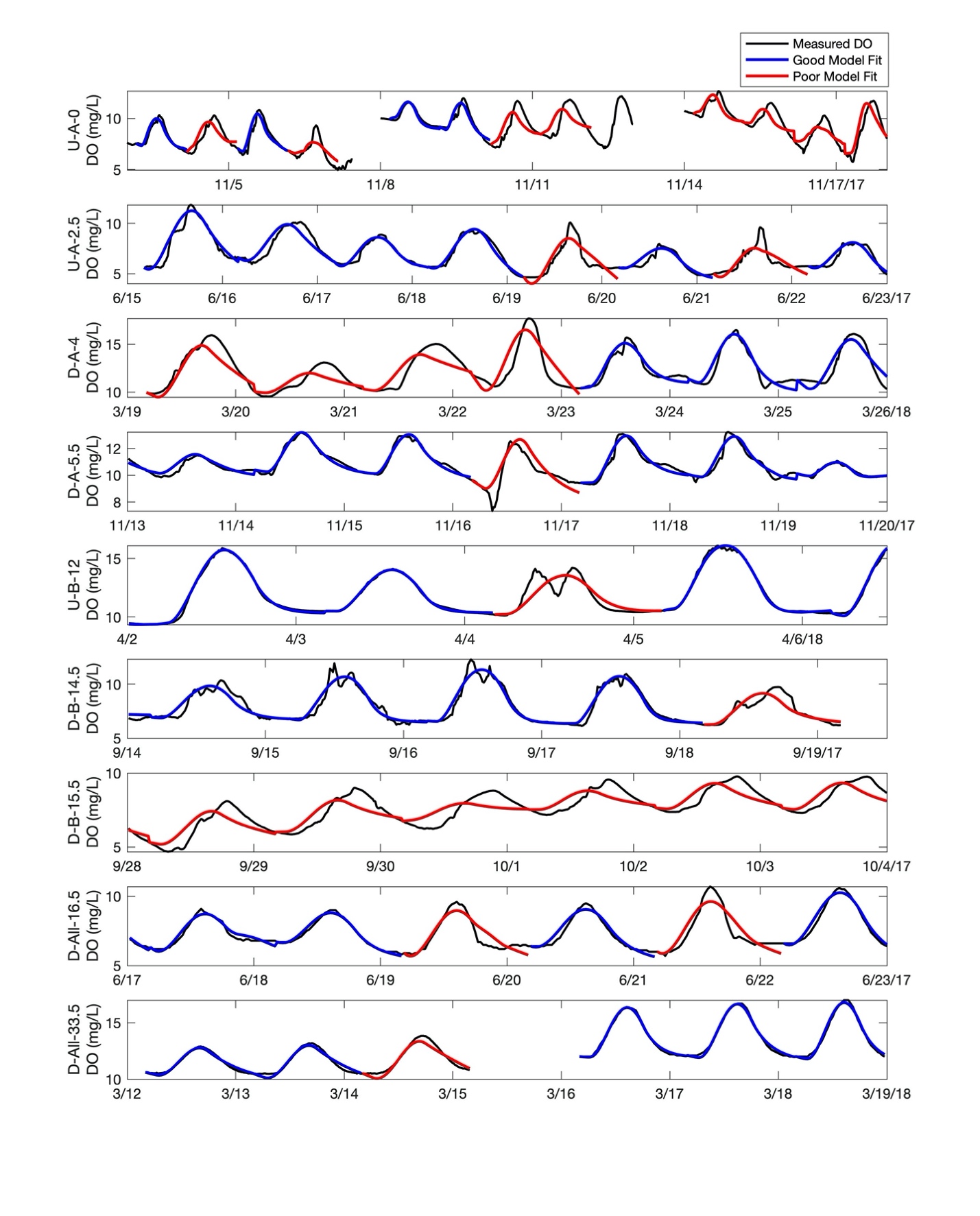


**S4 Fig. Examples of poor model fit.** Examples of modeling results from each site. The black line is the observed DO that was used as model input. Blue lines indicate the modeled DO signal for days that passed quantitative QAQC benchmarks and a visual inspection for fit. Red lines indicate modeled DO signal for days that pass the QAQC benchmarks but a visual inspection indicated fit between the model and observed data was poor. One example from each monitoring site is shown.

**S5 Fig. ER vs. K600.** ER vs. K600 at each site, with the R^2^ of the linear regression noted.


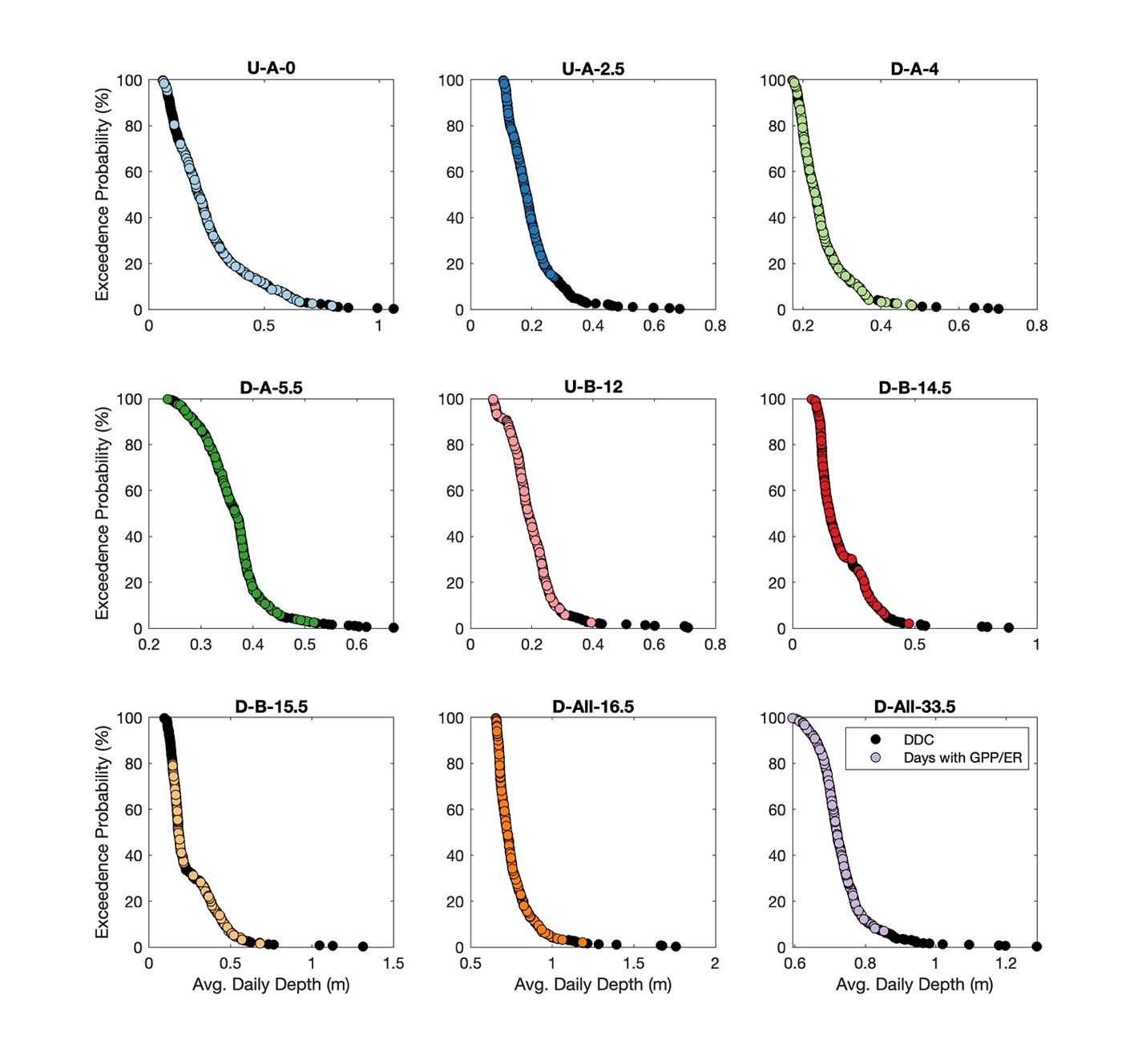


**S6 Fig. DDCs and modeled days.** Depth duration curves (DDC) for the period monitored, with all days in black and days with successful metabolism modeling in color. This indicates that, except for extremely high flows, we have measured metabolism over the range of flow at almost all the sites except U-A-0 and D-B-15.5.

**S7 Fig. Boxplots of seasonal NEP.** Boxplots of seasonal NEP rates by site from upstream (left) to downstream (right). All sites exhibited strong seasonal differences (p < 0.001) according to the Kruskal-Wallis rank sum test and results are shown with letters indicating differences among seasons.


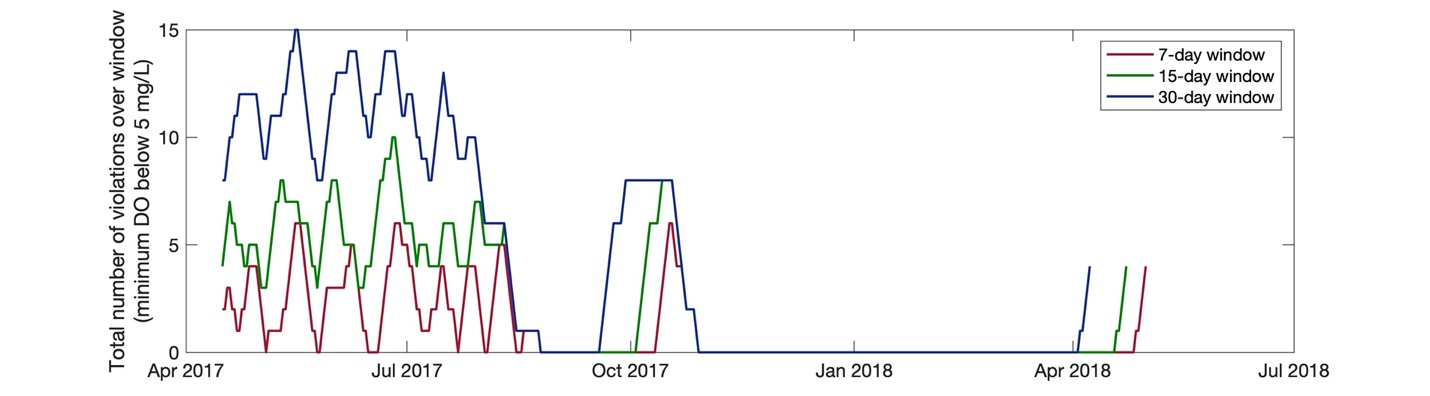


**S8 Fig. Sum of non-attainment days with different windows.** Number of days below 5 mg L^-1^ at U-A-0 summed over different sampling windows. During spring, most of the 30-day windows show 8 to 14 days below 5 mg/L. That drops to around 4 to 8 days for the 15-day window and only 1 to 4 days with a 7-day window.

**S9 Fig. DO storm response.** Hydrograph at D-All-16.5 and DO response at all loggers with a record during this time, recovering from the largest storm during the monitoring period.


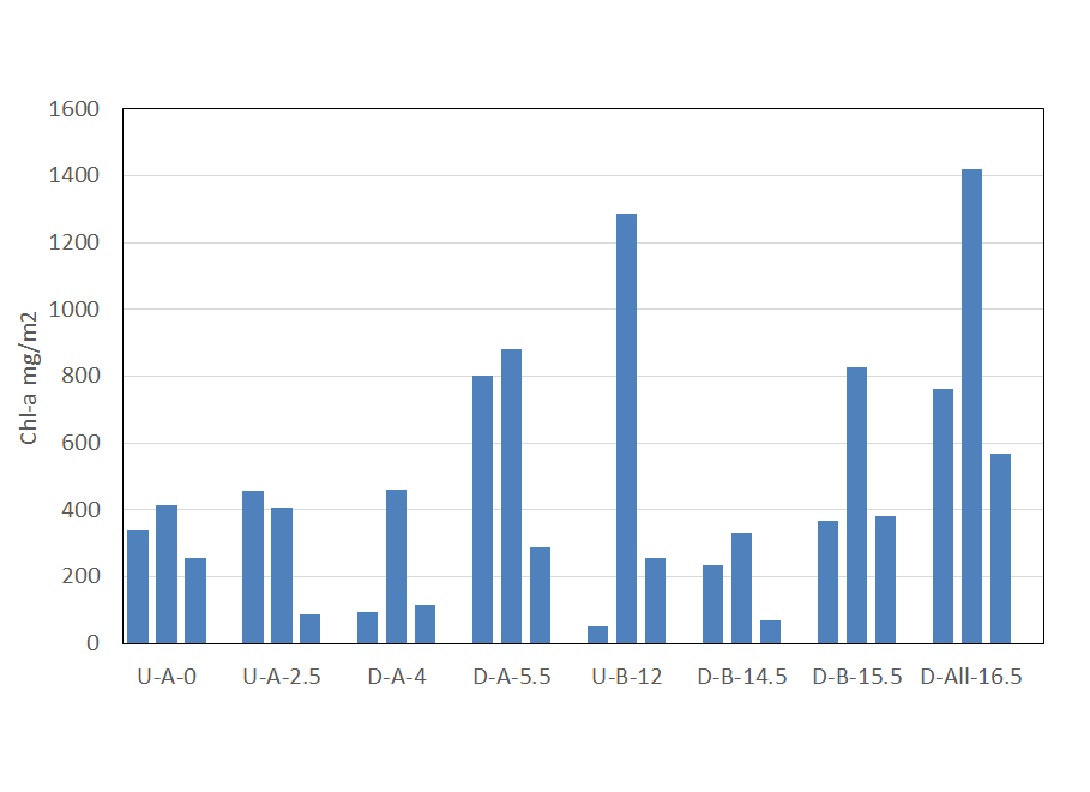


**S10 Fig. Benthic chlorophyll-a.** Benthic chlorophyll-a (Chl-a) collected at sites along a cross section (left bank, center, right bank). Sampling was conducted in April 2018 during dry weather before leaf out (no canopy cover). At each site, three rocks were sampled on each of the left bank, the center, and the right bank of the stream. These three rocks were composited so that at each site three samples were collected from three locations. The sample area was uniform (4 by 4 cm). The rocks were scraped with a wire brush and flushed with de-ionized water into a pre-weighted sample vial. DS-All-33.5 was not sampled. Samples were analyzed by the Academy of Natural Sciences of Drexel University.

**.Table S1: Kruskal-Wallis results for spring/fall**

|  | U-A-0 | U-A-2.5 | D-A-4 | D-A-5.5 | U-B-12 | D-B-14.5 | D-B-15.5 | D-All-16.5 | D-All-33.5 |
| --- | --- | --- | --- | --- | --- | --- | --- | --- | --- |
| U-A-0 | x | <0.0001 | 1 | <0.0001 | <0.0001 | 0.0011 | 0.74 | 1 | <0.0001 |
| U-A-2.5 | <0.0001 | x | <0.0001 | 0.21 | 1 | <0.0001 | 0.0077 | <0.0001 | 0.1413 |
| D-A-4 | 0.1073 | <0.0001 | x | <0.0001 | <0.0001 | 0.0003 | 0.89 | 0.97 | <0.0001 |
| D-A-5.5 | <0.0001 | 0.1073 | <0.0001 | x | 0.0186 | <0.0001 | 0.41 | <0.0001 | 1 |
| U-B-12 | <0.0001 | 0.2994 | <0.0001 | <0.0001 | x | <0.0001 | 0.0011 | <0.0001 | 0.0084 |
| D-B-14.5 | 0.0022 | <0.0001 | 0.109 | <0.0001 | <0.0001 | x | 0.0001 | 0.0009 | <0.0001 |
| D-B-15.5 | <0.0001 | 1 | <0.0001 | 0.2318 | 1 | <0.0001 | x | 0.36 | 0.41 |
| D-All-16.5 | 0.0967 | <0.0001 | 0.0008 | 0.0257 | <0.0001 | <0.0001 | 0.0004 | x | <0.0001 |
| D-All-33.5 | <0.0001 | 0.226 | <0.0001 | <0.0001 | 1 | <0.0001 | 1 | <0.0001 | x |

Table of p-values from Fig 3. Overall test results for GPP (above the x’s): p < 0.0001; chi-squared test statistic = 348.42; df = 1113. Overall test results for ER (below the x’s): p < 0.0001; chi-squared test statistic = 420.46; df = 1113

**Table S2: Kruskal-Wallis results for winter.**

|  | U-A-0 | U-A-2.5 | D-A-4 | D-A-5.5 | U-B-12 | D-B-14.5 | D-B-15.5 | D-All-16.5 | D-All-33.5 |
| --- | --- | --- | --- | --- | --- | --- | --- | --- | --- |
| U-A-0 | x | 1 | 1 | 0.9721 | 0.9685 | 0.0001 | 0.7474 | 0.0058 | 0.4843 |
| U-A-2.5 | 1 | x | 1 | 0.9738 | 0.9705 | 0.0016 | 0.9146 | 0.0383 | 0.5691 |
| D-A-4 | 0.4963 | 0.6936 | x | 1 | 1 | 0.0001 | 0.5763 | 0.0053 | 0.9145 |
| D-A-5.5 | 1 | 1 | 0.187 | x | 1 | <0.0001 | 0.0386 | <0.0001 | 0.9391 |
| U-B-12 | 0.0893 | 0.1873 | 0.0001 | 0.0379 | x | <0.0001 | 0.0398 | <0.0001 | 0.9544 |
| D-B-14.5 | 0.0034 | 0.024 | 0.9088 | <0.0001 | <0.0001 | x | 0.0335 | 1 | <0.0001 |
| D-B-15.5 | 1 | 1 | 0.4049 | 1 | 0.0754 | 0.0013 | x | 0.3995 | 0.0021 |
| D-All-16.5 | 0.0096 | 0.0501 | 0.9646 | 0.0002 | <0.0001 | 1 | 0.0043 | x | <0.0001 |
| D-All-33.5 | 0.0351 | 0.0837 | <0.0001 | 0.0144 | 1 | <0.0001 | 0.029 | <0.0001 | x |

Table of p-values from Fig 3. Overall test results for GPP (above the x’s): p < 0.0001; chi-squared test statistic = 94.09; df = 182. Overall test results for ER (below the x’s): p < 0.0001; chi-squared test statistic = 111.03; df = 182

**Table S3: Kruskal-Wallis results for spring 2018.**

|  | U-A-0 | U-A-2.5 | D-A-4 | D-A-5.5 | U-B-12 | D-B-14.5 | D-B-15.5 | D-All-16.5 | D-All-33.5 |
| --- | --- | --- | --- | --- | --- | --- | --- | --- | --- |
| U-A-0 | x | 0.5472 | 0.0139 | 0.2044 | 0.9875 | 0.0006 | 1 | <0.0001 | 1 |
| U-A-2.5 | 0.6965 | x | 0.0002 | 0.0042 | 0.1268 | 0.8728 | 0.3479 | 0.8334 | 0.777 |
| D-A-4 | 1 | 0.3504 | x | 0.9193 | 0.0802 | <0.0001 | 0.0726 | <0.0001 | 0.0005 |
| D-A-5.5 | 0.0242 | 0.0011 | 0.4466 | x | 0.6581 | <0.0001 | 0.561 | <0.0001 | 0.0144 |
| U-B-12 | 0.0025 | 0.0002 | 0.1844 | 1 | x | <0.0001 | 1 | <0.0001 | 0.6694 |
| D-B-14.5 | <0.0001 | 0.3225 | <0.0001 | <0.0001 | <0.0001 | x | 0.0002 | 1 | 0.0018 |
| D-B-15.5 | 1 | 0.824 | 0.9854 | 0.0161 | 0.0017 | 0.0001 | x | <0.0001 | 0.9736 |
| D-All-16.5 | <0.0001 | 0.6296 | <0.0001 | <0.0001 | <0.0001 | 0.9857 | <0.0001 | x | <0.0001 |
| D-All-33.5 | .0873 | 0.004 | 0.7444 | 1 | 0.9725 | <0.0001 | 0.059 | <0.0001 | x |

Table of p-values from Fig 3. Overall test results for GPP (above the x’s): p < 0.0001; chi-squared test statistic = 147.6; df = 416. Overall test results for ER (below the x’s): p < 0.0001; chi-squared test statistic = 219.06; df = 416

**Table S4. Kruskal-Wallis results for all dates.**

|  | U-A-0 | U-A-2.5 | D-A-4 | D-A-5.5 | U-B-12 | D-B-14.5 | D-B-15.5 | D-All-16.5 | D-All-33.5 |
| --- | --- | --- | --- | --- | --- | --- | --- | --- | --- |
| U-A-0 | x | <0.0001 | 0.8905 | <0.0001 | <0.0001 | <0.0001 | 0.53 | 0.035 | <0.0001 |
| U-A-2.5 | <0.0001 | x | 0.0002 | 1 | 1 | <0.0001 | 0.0397 | <0.0001 | 0.675 |
| D-A-4 | 0.6396 | <0.0001 | x | 0.0001 | <0.0001 | <0.0001 | 1 | 0.0001 | 0.029 |
| D-A-5.5 | <0.0001 | 1 | <0.0001 | x | 0.9547 | <0.0001 | 0.0406 | <0.0001 | 0.714 |
| U-B-12 | <0.0001 | <0.0001 | <0.0001 | <0.0001 | x | <0.0001 | 0.0016 | <0.0001 | 0.0626 |
| D-B-14.5 | <0.0001 | <0.0001 | <0.0001 | <0.0001 | <0.0001 | x | <0.0001 | 0.0044 | <0.0001 |
| D-B-15.5 | <0.0001 | 1 | <0.0001 | 1 | 0.0841 | <0.0001 | x | <0.0001 | 0.5515 |
| D-All-16.5 | 0.3621 | <0.0001 | 1 | <0.0001 | <0.0001 | <0.0001 | <0.0001 | x | <0.0001 |
| D-All-33.5 | <0.0001 | 0.0017 | <0.0001 | 0.0082 | 0.9749 | <0.0001 | 0.4124 | <0.0001 | x |

Table of p-values from Fig 3. Overall test results for GPP (above the x’s): p < 0.0001; chi-squared test statistic = 387.98; df = 1789. Overall test results for ER (below the x’s): p < 0.0001; chi-squared test statistic = 565.43; df = 1789

**S5 Table. Statistical results from seasonal comparison.**

| Site | GPP | | | ER | | | NEP | | |
| --- | --- | --- | --- | --- | --- | --- | --- | --- | --- |
|  | p-value | KW chi-squared | df | p-value | KW chi-squared | df | p-value | KW chi-squared | df |
| U-A-0 | <0.001 | 34.98 | 152 | <0.001 | 48.51 | 152 | <0.001 | 61.76 | 152 |
| U-A-2.5 | <0.001 | 81.81 | 177 | <0.001 | 37.64 | 177 | <0.001 | 58.91 | 177 |
| D-A-4 | <0.001 | 25.79 | 133 | <0.001 | 30.81 | 133 | <0.001 | 36.24 | 133 |
| D-A-5.5 | <0.001 | 80.2 | 252 | <0.001 | 71.84 | 252 | <0.001 | 85.8 | 252 |
| U-B-12 | <0.001 | 110.99 | 262 | <0.001 | 63.39 | 262 | <0.001 | 131.3 | 262 |
| D-B-14.5 | <0.001 | 38.31 | 118 | <0.001 | 33.27 | 118 | <0.001 | 31.4 | 118 |
| D-B-15.5 | <0.001 | 26.89 | 88 | <0.001 | 41.06 | 88 | 0.084 | 4.95 | 88 |
| D-All-16.5 | <0.001 | 130.08 | 306 | <0.001 | 137.4 | 306 | <0.001 | 68.48 | 306 |
| D-All-33.5 | <0.001 | 82.24 | 293 | <0.001 | 52.93 | 293 | <0.001 | 70.73 | 293 |

Statistical results from Kruskal-Wallis tests comparing metabolic rates across seasons at each site, showing in Fig 6 (GPP and ER) and S7 Fig (NEP).

**S6 Table. Windowing results.**

| Site | min. length (d) | med. length (d) | mean length (d) | max. length (d) | Minimum DO exceedances (d) |
| --- | --- | --- | --- | --- | --- |
| U_A_0 | 9 | 41 | 45 | 84 | 59 |
| U_A_2.5 | 14 | 29 | 41 | 76 | 37 |
| D_A_4 | 1 | 16 | 21 | 54 | 49 |
| D_A_5.5 | 5 | 56 | 53 | 93 | 3 |
| U_B_12 | 1 | 32 | 54 | 143 | 3 |
| D_B_14.5 | 2 | 13 | 14 | 32 | 12 |
| D_B_15.5 | 1 | 14 | 25 | 71 | 0 |
| D_All_16.5 | 3 | 106 | 129 | 279 | 5 |
| D_All_33.5 | 3 | 96 | 90 | 162 | 0 |

Distribution of consecutive days with measurements and number of days that were modeled that also had minimum DO exceedances (>5 mg L^-1^).
